# Comparing the dietary niche overlap and ecomorphological differences between invasive *Hemidactylus mabouia* geckos and a native gecko competitor

**DOI:** 10.1002/ece3.8401

**Published:** 2021-12-20

**Authors:** April D. Lamb, Catherine A. Lippi, Gregory J. Watkins‐Colwell, Andrew Jones, Dan L. Warren, Teresa L. Iglesias, Matthew C. Brandley, Alex Dornburg

**Affiliations:** ^1^ Department of Bioinformatics and Genomics University of North Carolina Charlotte North Carolina USA; ^2^ North Carolina Museum of Natural Sciences Raleigh North Carolina USA; ^3^ Department of Applied Ecology North Carolina State University Raleigh North Carolina USA; ^4^ Department of Geography Quantitative Disease Ecology and Conservation (QDEC) Lab Group University of Florida Gainesville Florida USA; ^5^ Division of Vertebrate Zoology Yale Peabody Museum of Natural History New Haven Connecticut USA; ^6^ Department of Ecology and Evolutionary Biology Yale University New Haven Connecticut USA; ^7^ Biodiversity and Biocomplexity Unit Okinawa Institute of Science and Technology Graduate University Onna, Kunigami District Okinawa Prefecture Japan; ^8^ Animal Resource Section Okinawa Institute of Science and Technology Graduate University Onna, Kunigami District Okinawa Prefecture Japan; ^9^ Section of Amphibians and Reptiles Carnegie Museum of Natural History Pittsburgh Pennsylvania USA

**Keywords:** food web, invasive species, trophic ecology, urbanization, vertebrate biodiversity loss

## Abstract

*Hemidactylus mabouia* is one of the most successful, widespread invasive reptile species and has become ubiquitous across tropical urban settings in the Western Hemisphere. Its ability to thrive in close proximity to humans has been linked to the rapid disappearance of native geckos. However, aspects of *Hemidactylus mabouia* natural history and ecomorphology, often assumed to be linked with this effect on native populations, remain understudied or untested. Here, we combine data from ∂15N and ∂13C stable isotopes, stomach contents, and morphometric analyses of traits associated with feeding and locomotion to test alternate hypotheses of displacement between *H*. *mabouia* and a native gecko, *Phyllodactylus martini*, on the island of Curaçao. We demonstrate substantial overlap of invertebrate prey resources between the species, with *H*. *mabouia* stomachs containing larger arthropod prey as well as vertebrate prey. We additionally show that *H*. *mabouia* possesses several morphological advantages, including larger sizes in feeding‐associated traits and limb proportions that could offer a propulsive locomotor advantage on vertical surfaces. Together, these findings provide the first support for the hypotheses that invasive *H*. *mabouia* and native *P*. *martini* overlap in prey resources and that *H*. *mabouia* possess ecomorphological advantages over *P*. *martini*. This work provides critical context for follow‐up studies of *H*. *mabouia* and *P*. *martini* natural history and direct behavioral experiments that may ultimately illuminate the mechanisms underlying displacement on this island and act as a potential model for other systems with *Hemidactylus mabouia* invasions.

## Graphical abstract

*Hemidactylus mabouia* is one of the most successful, widespread invasive reptile species and has become ubiquitous across tropical urban settings in the Western Hemisphere. Its ability to thrive in close proximity to humans has been linked to the rapid disappearance of native geckos. However, the mechanisms driving this phenomenon remain understudied. Here, we combine data from ∂15N and ∂13C stable isotopes, stomach contents, and morphometric analyses of traits associated with feeding and locomotion to test for the presence of ecological and morphological overlap between *H. mabouia* and a declining native gecko, *Phyllodactylus martini*, on the Lesser Antillean Island of Curaçao. Through diet content and isotopic analyses, we demonstrate substantial overlap of invertebrate prey resources between the two species. Additionally, we find that *H. mabouia* possess several morphological advantages over *P. martini*, including larger sizes in feeding‐associated traits and limb proportions that could offer a propulsive locomotor advantage on vertical surfaces. Our results provide critical baseline evidence of dietary and morphological overlap between the two species. Furthermore, the morphological advantages of *H. mabouia* are noteworthy, but warrant the need for future investigation of functional locomotor morphology and performance of these species to better discern the exact mechanisms through which *H. mabouia* may be displacing *P*. *martini*.

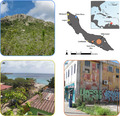

## 1 INTRODUCTION

*Hemidactylus mabouia* (Tropical House Gecko) is perhaps the most pervasive and formidable gecko to invade the Western Hemisphere (Agarwal et al., 2021; Weterings & Vetter, 2018). This species will readily capitalize on the aggregation of insects around human light sources (Hughes et al., 2015), a foraging strategy which promotes high densities of individual *H*. *mabouia* that use aggressive tactics to restrict access to these spatially clustered food resources (Short & Petren, 2012; van Buurt, 2004; Williams et al., 2016). While this suggests competition to be an important aspect of *H*. *mabouia* invasions, dietary niche overlap with native species is often not known, and other hypotheses concerning possible ecological advantages over native species remain little explored. For example, the feeding mode of *H*. *mabouia* combines ambush tactics (Vitt, 1983) with active pursuit of nearby prey (Dornburg et al., 2016). Such a foraging mode could have been selective for larger hind limb or shorter fore‐limb proportions that, respectively, offer a locomotor advantage over native species when accelerating or decelerating on sheer vertical surfaces common in urbanizing landscapes (Zaaf & Van Damme, 2001). Given that both dietary overlap and morphological advantages have been invoked as major drivers of displacement in the wake of invasions by the distantly related *Hemidactylus frenatus* in the Pacific (Bolger & Case, 1992; Case et al., 1994; Petren & Case, 1996, 1998; Short & Petren, 2012), case studies from across the range of *H*. *mabouia* are warranted if we are to gain a broader understanding of the factors that promote the success of *H*. *mabouia* invasions.

The gradual decline of the native *Phyllodactylus martini* (Dutch Leaf Tailed Gecko) from urbanizing areas on the Lesser Antillean island of Curaçao provides an exceptional opportunity for developing further hypotheses of what allows *H*. *mabouia* to replace ecologically similar geckos. Similar to *H*. *mabouia*, *P*. *martini* readily colonizes walls and takes advantage of insect and arachnid prey drawn to artificial lights (Dornburg et al., 2016; van Buurt, 2004). Although *P*. *martini* is predicted to fare well in suburban conditions (Dornburg et al., 2016; van Buurt, 2004), established populations have been rapidly declining following the introduction of *H*. *mabouia* in the 1980s (Dornburg et al., 2016; Hughes et al., 2015; van Buurt, 2004, 2006). This decline has been attributed to *H*. *mabouia* possessing a superior competitive ability based primarily on behavioral observations (Hughes et al., 2015), with no quantification of dietary overlap between these species. Therefore, it is unclear if there are differences in morphological traits relevant to foraging between *H*. *mabouia* and *P*. *martini*.

Variation in locomotor morphology and size plays vital roles in the feeding ecology of lizards. However, a quantitative comparison of these traits between *H*. *mabouia* and *P*. *martini* have yet to be conducted. Analyses of limb lengths relevant to acceleration or deceleration on vertical surfaces would provide evidence for whether *H*. *mabouia* possess locomotor traits that enable more efficient prey pursuit and capture. Likewise, head size represents another key ecological trait for investigating the invasion biology of these species. Increased head sizes are correlated with more efficient prey capture in lizards (Dufour et al., 2018; Verwaijen et al., 2002). On the one hand, if aspects of *H*. *mabouia* cranial morphology are larger than those in *P*. *martini*, this could confer an advantage to energy‐efficient prey capture for *H*. *mabouia*. On the other hand, changes in head size that are correlated with shifts in prey would suggest a segregation of dietary niches, illuminating a potential mechanism of coexistence. Collectively, testing both the degree of dietary niche and ecomorphological overlap between these two species would improve our understanding of the invasion dynamics of *H*. *mabouia*. Moreover, this study provides a comparative framework to test hypotheses that drive the evolutionary success of introduced *H*. *mabouia* populations worldwide.

Here, we quantify dietary niche and morphological overlap between the invasive *Hemidactylus mabouia* and native *Phyllodactylus martini* geckos on Curaçao. We focus on three ecological aspects that may underlie the disappearance of *P*. *martini* following the establishment of *H*. *mabouia*. (1) We test expectations of dietary niche overlap between the two species by quantifying levels of isotopic trophic signatures and prey overlap using analyses of nitrogen and carbon stable isotopes and direct examination of stomach contents. Additionally, we test for differences in (2) limb measurements associated with sprinting speed and deceleration on vertical surfaces, and (3) head size differences that both convey a prey capture advantage. These morphological analyses allowed us to test the hypothesis that *H*. *mabouia* possesses advantages over its potential native competitor in morphological traits associated with feeding and locomotion. Combined, our results cast new light on hypothesized mechanisms of *P*. *martini* displacement in urbanizing habitats following *H*. *mabouia* introductions on Curaçao.

## 2 MATERIALS AND METHODS

### 2.1 Fieldwork and data acquisition

**FIGURE 1:**
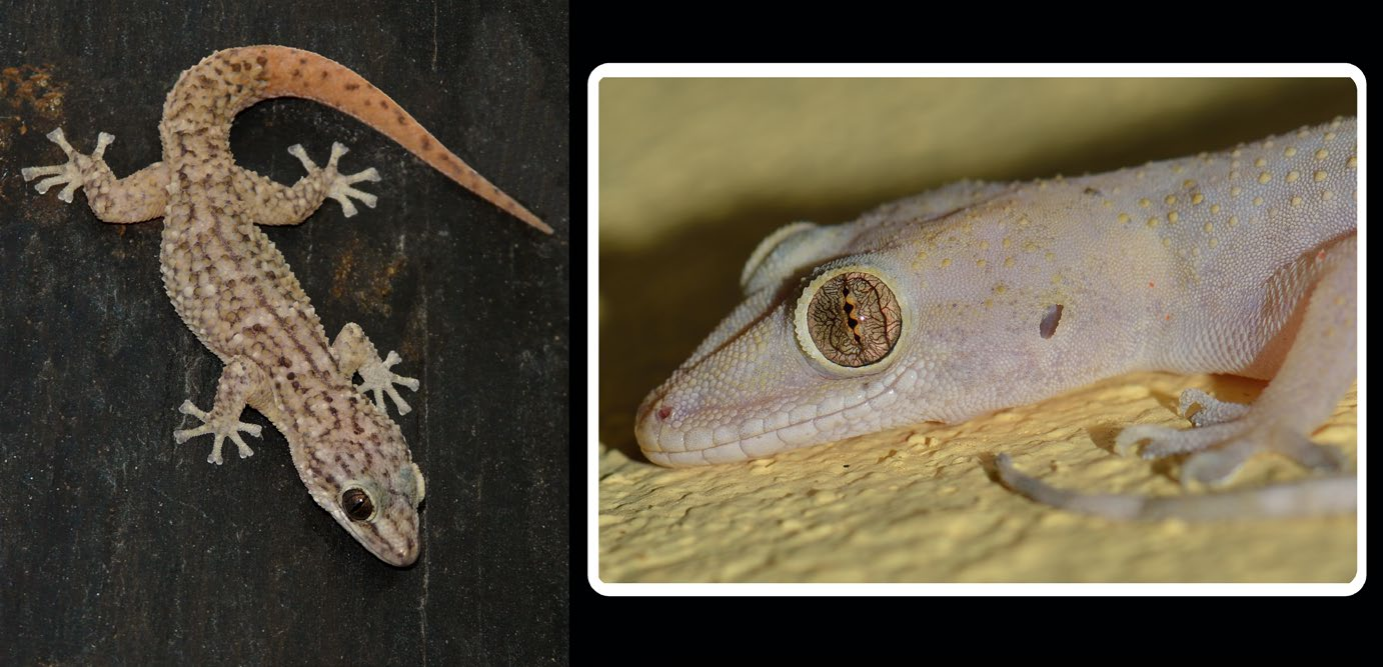
Image of a Dutch Leaf Toed Gecko – *Phyllodactylus martini* (left side) – and a Tropical House Gecko – *Hemidactylus mabouia* (right side) – in the Lesser Antilles. *P*. *martini* image modified from an image by Maarten Gilbert published under a Creative commons license BY‐NC‐ND. Image of *Hemidactylus mabouia* modified from an image taken by Gerard van Buurt under a Creative commons license BY‐NC‐ND. Both images are available at www.dutchcaribbeanspecies.org

**FIGURE 2:**
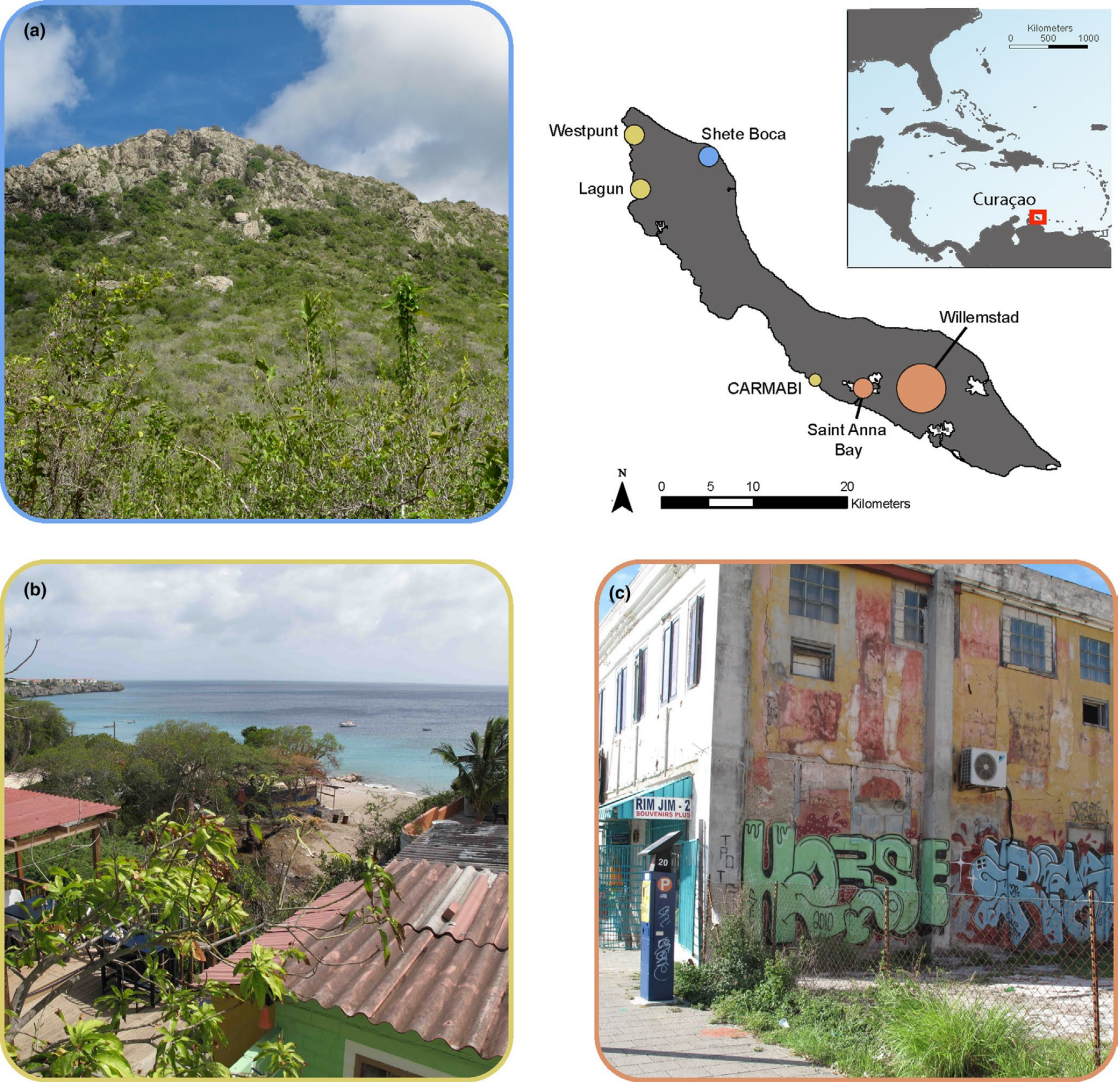
Map of sampling locations and representative images of habitats. Location and map of the island of Curaçao. Circles indicate sampling locations and species sympatry. (a) Sympatric area (yellow circles). (b) Allopatric “mondi” habitat with no *Hemidactylus mabouia* presence (blue circle). (c) Allopatric urban habitat with no *Phyllodactylus martini* presence (pink circles)

*Hemidactylus mabouia* (*n* = 90) and *Phyllodactylus martini* (*n* = 71) specimens (Figure 1) were collected at six sites across Curaçao between July 2009 and September 2011: Lagun, Westpunt, CARMABI (Caribbean Research and Management of Biodiversity foundation), Shete Boca, Saint Anna Bay, and Willemstadt (Table S1; Figure 2). Habitat type and species occupancy vary across sampling locations. For example, both species co‐occur in Lagun and Westpunt. At these sites, we restricted our sampling to suburban areas near natural habitats to maximize the potential of both species co‐occurring as *P*. *martini* has been found to be absent far from edge habitats in the presence of *H*. *mabouia* (Hughes et al., 2015). In contrast, Shete Boca is a natural area in which *H*. *mabouia* are absent, while Saint Anna Bay and Willemstadt are urban areas in which *P*. *martini* are absent. Across sites, sample locations included walls, rocks, outcrops, trees, thatch roofs, open ground, and shrubbery. Specimens were euthanized using MS‐222 within 30 min of capture (Conroy et al., 2009). Prior to preservation, muscle biopsies were taken from each individual and dehydrated for analysis of stable isotopes. Additionally, leaf samples from each locality were collected and dehydrated for use as baselines in isotopic analyses. Specimens were then fixed in 10% formalin and later transferred to 70% ethanol and deposited in the Yale Peabody Museum of Natural History (Table S1).

Adult *H*. *mabouia* (*n* = 30) and *P*. *martini* (*n* = 48) specimens collected in 2011 had their stomach contents preserved in 10% formalin and dissected, with contents identified and enumerated under a dissecting MVX10 microscope (Olympus Corp.; http://www.olympus‐lifescience.com/). During dissection, the sex of each individual was determined by visual inspection of the gonads. Prey items were identified to the taxonomic groupings similar to those in other studies of Caribbean lizards (e.g., Perry, 1996): *Arachnida* (*scorpiones*), *Arachnida* (*Araneae*), *Blattaria* (*Blattodea*), *Chilopoda*, *Coleoptera*, *Diptera*, *Ephemeroptera*, *Hemiptera*, *Hymenoptera*, *Isopoda*, *Lepidoptera*, *Orthoptera*, and “other” for unidentifiable digested fragments. Any vertebrate remains encountered were additionally identified to the highest taxonomic resolution possible, and we additionally identified any parasites encountered in the stomach. As formalin and alcohol preservation can have heterogeneous effects on the volume of invertebrate organisms (Donald & Paterson, 1977), enumeration of diet contents was restricted to the relative frequency. For each species, the relative frequency of each prey item was calculated based on the total number of prey items encountered across all individuals of that species within a given set of sites (i.e., sympatric sites, Table S1; Figure 2). We further collected measurement data on 79 *H*. *mabouia* for 10 morphological traits associated with feeding and locomotion: snout–vent length (SVL), postorbital width, temporalis width, head length (distance between anterior margin of tympanum to the tip of the rostrum), jaw length (distance between posterior margin of last supralabial scale to the tip of the rostrum), head height (distance between the ventral and dorsal surfaces of the head at the eye), brachium length, antebrachium length, thigh length, and shin length. All measurements were taken to the nearest 0.01 mm using digital calipers (Fowler Promax). Both stomach content and morphological data were integrated with the dataset of Dornburg et al. (2016) who previously measured *Phyllodactylus martini* specimens for the same morphological traits (*n* = 34; Zenodo https://doi.org/10.5281/zenodo.61569) and prey items (*n* = 69; Zenodo https://doi.org/10.5281/zenodo.61569).

### 2.2 Stable isotopic analysis of trophic ecology

Leg muscle biopsies from 21 individual *Hemidactylus mabouia* and 17 *Phyllodactylus martini* as well as 8 plant stems and leaf baseline samples were used in nitrogen (∂15N) and carbon (∂13C) stable isotope analysis. Although tail tips are commonly used in lizard isotope studies (Delibes et al., 2015), variation in lipid storage within gecko tails has the potential to confound analysis (Rode et al., 2016). Therefore, leg tissue was chosen as an alternative that also provided consistent yields of tissue required for analysis. Skin was removed from each muscle biopsy, and individual muscle and plant baseline samples were dehydrated at 40°C degrees for 48 h. Following dehydration, samples were powdered using a bead beater (MP FastPrep24 Hyland Scientific). From each sample, 1.5 mg of powder was loaded into 3 × 5 mm tins. Samples were analyzed at the University of California Davis Stable Isotope Facility using an isotope ratio mass spectrometer. As nitrogen enrichment can vary over spatial or temporal periods, quantification of trophic position for each individual was standardized using primary producer baseline samples from plant leaves and stems collected at each locality (Des Roches et al., 2016; Vidal & Sabat, 2010). To account for ∂15N values not reflecting primary producer‐level values (Marshall et al., 2007), baseline samples were compared across sites with aberrant samples (i.e., primary producer ∂15N > consumer ∂15N) removed. Nitrogen values were standardized following Post (2002), in subtracting the mean ∂15N of the primary producers from ∂15N of each individual lizard and assuming fractionation of 3.4% per trophic level (Post, 2002). ∂15N values for each species were visualized using raincloud plots (Allen et al., 2019). We tested for differences between the mean ∂15N values of *H*. *mabouia* and *P*. *martini* using a Welch's *t*‐test and additionally used Levene's test to assess whether there was a significant difference in ∂15N variance between species. To test for potential differences in ∂13C, we used the same statistical approaches as those used in the analysis of ∂15N, assuming carbon fractionation to be 0% (Post, 2002). In this case, non‐significant differences in ∂13C would support the expectation that these species forage in similar habitats. To reduce the likelihood of a Type 1 error, p‐values for all analyses involving multiple comparisons were adjusted using a Benjamini–Hochberg (BH) procedure (*p*_adjusted_ = *q*; Thissen et al., 2002). All analyses were conducted in R, v. 3.4.3 (R Development Core Team, 2018).

### 2.3 Stomach content analysis

Stomach contents were analyzed to test for dietary niche overlap between species that would support a hypothesis of resource competition. Differences in stomach contents between species were visualized using a principal components analysis. Relative frequencies were calculated as stated above and compared between species in sympatry and also within species between sympatric and allopatric sites. This allowed us to assess if *P*. *martini* is altering its prey base in urbanizing settings where it co‐occurs with *H*. *mabouia*, and also to visualize dietary niche overlap. To test for significant differences between species, we first used an analysis of similarity (ANOSIM; Chapman & Underwood, 1999; Clarke, 1993) based on Bray–Curtis distances and 999 permutations in the vegan software package (Oksanen, 2011; Oksanen et al., 2007). Differences in mean ranks were quantified using the R statistic, for which values close to 0 indicating high similarity and values close to 1 indicating high dissimilarity (Chapman & Underwood, 1999). Additionally, we quantified the Schoener's D index (*α*) to assess dietary overlap between species when these co‐occur with values close to 0 indicating little dietary overlap and values close to 1 indicating high dietary overlap (Schoener, 1974). A threshold of 0.60 was used to determine significant overlap (Wallace, 1981). Stomach content data were pooled from sites where species co‐occur, as visualizations of dietary overlap indicated similar patterns of dietary overlap and prey consumption between sites. We also tested for differences within species by comparing their dietary overlap in sympatry versus allopatry. Males and females were pooled; Dornburg et al. (2016) previously found no significant dietary differences between *P*. *martini* males and females using these data, so only *H*. *mabouia* males and females were compared. As an ANOSIM analysis can be misled when small sample sizes are coupled with high dispersion between samples (Anderson & Walsh, 2013), these were not conducted in instances where sample sizes were lower than 20. Given that internal parasites are common in geckos (see review in Dornburg et al., 2019), we tested for differences in nematode prevalence between species using a Welch's *t*‐test.

### 2.4 Comparisons of morphology

We compared absolute differences in log snout–vent length (SVL) between species using an ANOVA and created raincloud plots (Allen et al., 2019) to visualize differences. These plots combine classic boxplots with violin raw data plots to simultaneously visualize data, the difference in size quartiles, and a kernel density smoothed estimate of the frequency distribution of the SVL data. We conducted a principal components analysis (PCA) to visualize the overall morphospace occupied by both species. In geckos, size has been shown to covary with our target morphological measurements (Dornburg et al., 2016). To account for this, we first regressed all the measurements per species against log‐transformed SVL and used the residual values of individual traits regressed against log‐transformed SVL as data for the PCA. To assess if differences in morphospace occupancy were mostly driven by uneven sample sizes, we randomly sampled equal numbers of both gecko species from our data 200 times across a series of datasets that reflect a range of sample sizes. We resampled our data to assemble a series of datasets that span intervals of 5 additional samples per species starting with 10 and ending with 55 individuals per species. For each of these 2000 datasets, we conducted a PCA and computed the mean and quantiles (25% and 75%) of the ratio of *H*. *mabouia*‐to‐*P*. *martini* morphospace.

While morphospace visualization is advantageous for assessing the overall overlap of phenotypic variation, it is possible that allometric slopes are identical between species and simply have different intercepts (i.e., at a given body size, a focal trait in one species is larger in one species than the other). To further scrutinize our data, we used an analysis of covariance (ANCOVA) to test for differences in each morphological trait between species. For each analysis, we kept the log‐transformed SVL as the covariate and treated each log‐transformed morphometric measurement (e.g., jaw length, limb length, etc.) as the response. This approach allowed us to test the potential correlation for each measured trait and SVL as well as the possibility of significant differences between species that take trait covariation with SVL into account. We repeated analyses with non‐significant interactions removed, as inclusion or omission of non‐significant interactions can potentially impact ANCOVA analyses.

In many lizard species, including geckos, head size is a sexually dimorphic trait with males often having larger heads relative to females (Iturriaga & Marrero, 2013; Kratochvíl et al., 2002; Scharf & Mieri, 2013). Therefore, we used an ANCOVA to assess whether morphological differences for each trait were potentially masked when pooling sexes by species. For all analyses, we again kept the log‐transformed SVL as the covariate and treated each log‐transformed morphometric measurement as the response. Finally, we assessed potential differences in total limb lengths (brachium length + antebrachium length; thigh length + shin length) between species and sexes using log‐transformed limb length as the response and log‐transformed SVL as the covariate in an ANCOVA. This additional analysis facilitated additional comparisons of expectations of gecko locomotion as studies often discuss differences in total limb lengths.

Prior work has suggested large hind limbs compensate for large heads in the locomotion of *Hemidactylus* spp. geckos (Cameron et al., 2013). To examine scaling relationships between head size and hind limb length for both species, we constructed a set of generalized linear models (GLMs) using sex, species, SVL, and head size as explanatory variables, with one set of models using head length to quantify head size and another set using postorbital width. All models except the intercept‐only null models contained an interaction term between SVL and the head size term, so that the effects of head size on limb length would be controlled for overall body size. Additional candidate models included (1) sex, (2) species identity, and (3) sex, species identity, and an interaction term between species identity and head size. Model fit was evaluated with the Akaike information criterion with a correction for small sample size (AICc). This method of model selection identifies models that predict the data well while penalizing overparameterization (Burnham & Anderson, 2004).

## 3 RESULTS

### 3.1 Differences in feeding ecology

**FIGURE 3:**
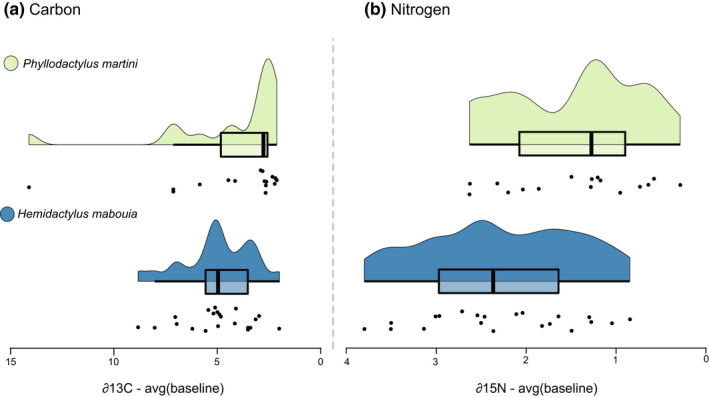
Raincloud plots of isotopic data. (a) Raincloud plots visualizing estimated trophic position for *Phyllodactylus martini* (*n* = 17) and *Hemidactylus mabouia* (*n* = 21) using Carbon and (b) Nitrogen. Raw carbon and nitrogen isotopic values (black circles) were corrected using average baseline values across all sites

Analysis of ∂15N revealed a significantly (Welch's *t*‐test: *p* < .004, *q* = 0.007, *t* = 3.123, df =34.272) higher mean trophic level in *Hemidactylus mabouia* versus *Phyllodactylus martini* (Figure 3a). In contrast, analysis of ∂13C isotopes revealed no significant (Welch's *t*‐test: *p* < .401, *q* = 0.401, *t* = 0.857, df = 21.812) difference in mean ∂13C between *H*. *mabouia* versus *P*. *martini* (Figure 3b), supporting the expectation that these two species overlap in major foraging habitat type. Levene's tests did not support a significant increase in ∂15N variance within *H*. *mabouia* versus *P*. *martini* (*F* = 0.480; *p* = .493, *q* = 0.493), although a single *P*. *martini* outlier point in our analysis depicted a carbon signature consistent with marine prey resource use, suggesting the possibility that some individuals may opportunistically forage close to the shoreline. Regardless, the difference in variance of ∂13C values between species was found to be non‐significant (*F* = 0.585; *p* = .449, *q* = 0.493), even after removing this potential outlier point (*F* = 0.056, *p* = .814, *q* = 0.814).

**FIGURE 4:**
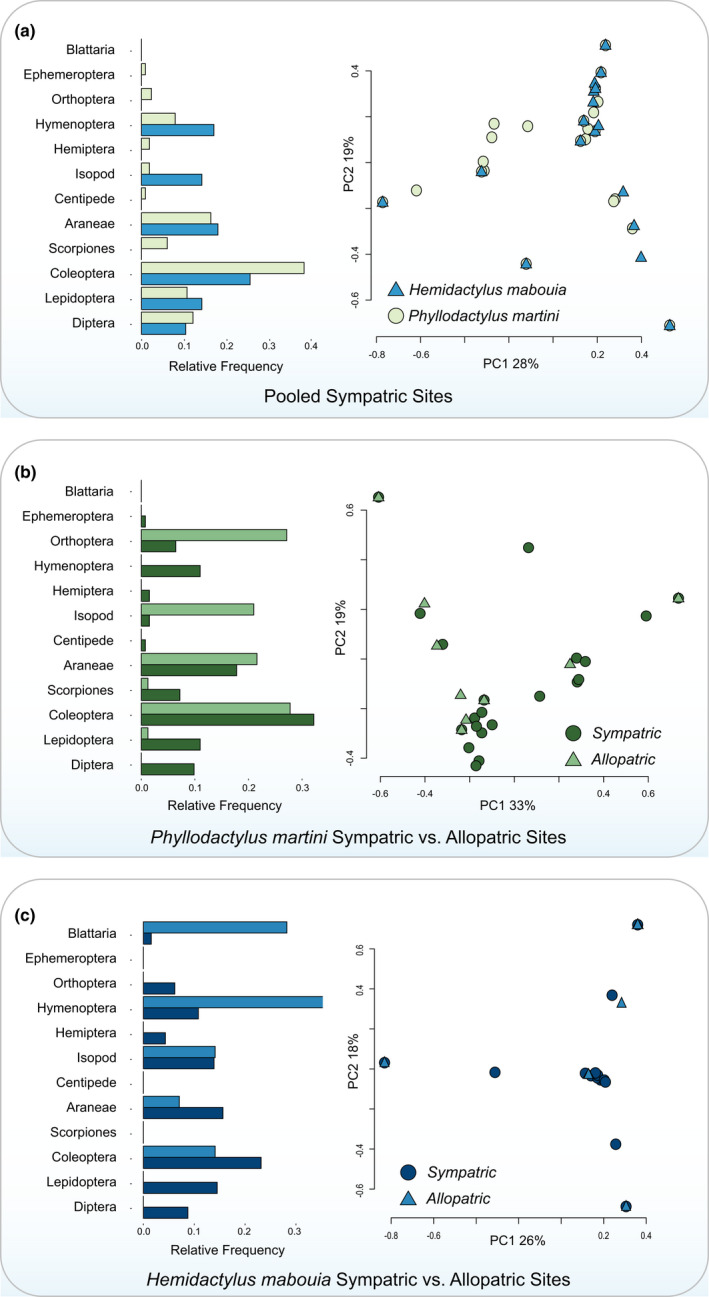
Relative frequency of prey items and principal component analysis contrasting stomach contents of *Phyllodactylus martini* (*n* = 79) and *Hemidactylus mabouia* (*n* = 57) (a) Pooled stomach contents by species at sympatric sites where species co‐occur. (b) Stomach contents of *P*. *martini* across sites where it co‐occurs (sympatric) or not (allopatric) with *Hemidactylus mabouia*. (c) Stomach contents of *H*. *mabouia* across sites where it co‐occurs (sympatric) or not (allopatric) with *P*. *martini*

Our analyses of individual stomach contents revealed *H*. *mabouia* to generally have fewer prey items per stomach than *P*. *martini* (Welch's *t*‐test: *p* < .001, *t* = 3.31, df = 84.74). Across 59 specimens of *H*. *mabouia*, we found 0 to 3 prey items per individual that collectively spanned a wide range of invertebrates. Additionally, three individual *H*. *mabouia* each contained a single vertebrate prey item. These prey items were identified as *Gonatodes antillensis*, *Phyllodactylus martini*, and *Ramphotyphlops braminus*. All instances of vertebrate predation by *H*. *mabouia* were found in areas where the two species overlap (Dornburg et al., 2011). Comparing the invertebrate prey found in *H*. *mabouia* to *P*. *martini* revealed the two species to consume similar prey items, but differ in the overall percentages of prey items consumed (Figure 4; Table S2). In comparing areas where the two species are sympatric, we found a significant Schoener index (*α* = 0.789) that supported a high degree of dietary niche overlap. The results of our PCA also supported a large degree of overlap in dietary niche space across all pooled samples (Figure 4a; Table 1), as well as at the site level (Figure S1). ANOSIM results supported a marginally significant difference in *P*. *martini's* dietary niche between suburban areas of overlap with *H*. *mabouia* when compared to the allopatric population at Shete Boca. This result was not robust to p‐value adjustment (*R* = 0.1475, *p* = .047, *q* = 0.094), although an *α* of 0.56 and a shift in dietary niche space (Figure 4b; Table 2) suggest *P*. *martini* may consume higher numbers of orthopterans and isopods when not in sympatry with *H*. *mabouia*. *H*. *mabouia* appears to also have a slightly broader dietary niche in areas of sympatry based on an *α* of 0.48 and visualization of dietary niche overlap (Figure 4c; Table 3), although limited power due to small sample sizes precludes confidence in a negative ANOSIM result (*R* = 0.03088, *p* = .201, *q* = 0.201). Similar to (Dornburg et al., 2016), we found *H*. *mabouia* males and females to broadly overlap in diet (Figure S2). In addition to prey contents, parasitism infestations by nematodes were significantly different between the two species (Welch's *t*‐test: *p* < .001, *t* = −3.768, df = 71), suggesting higher parasite pressure within *P*. *martini*.

**TABLE 1:** Principal component factor loadings by axis for invertebrate diet components of *Phyllodactylus martini* and *Hemidactylus mabouia* in sympatry

Prey	PC1|PrC	PC2|PrC	PC3|PrC
*Orthoptera*	0.066|0.038	0.042|0.021	0.050|0.029
*Araneae*	0.404|0.234	**−0.805**|**0.390**	−0.077|0.044
*Blattaria*	0.007|0.004	0.017|0.008	−0.022|0.013
*Chilopoda*	0.004|0.002	0.005|0.003	0.002|0.001
*Coleoptera*	**−0.896**|**0.519**	−0.267|0.129	−0.040|0.023
*Diptera*	0.015|0.001	0.087|0.042	−0.024|0.014
*Ephemeroptera*	0.004|0.003	0.008|0.004	0.013|0.007
*Hemiptera*	0.022|0.013	0.041|0.020	−0.029|0.017
*Hymenoptera*	0.093|0.093	0.299|0.145	**0.712**|**0.411**
*Isopoda*	0.081|0.047	0.067|0.033	0.064|0.037
*Lepidoptera*	0.113|0.065	0.418|0.202	−0.690|0.399
*Scorpiones*	0.024|0.014	0.008|0.004	0.011|0.006
% Variance explained	**16.66%**	**11.31%**	**8.85%**

Note

Top contributing components indicated in bold for each axis.

Abbreviations: PrC, Proportional contribution to PC axis.

**TABLE 2:** Principal component factor loadings by axis for invertebrate diet components of *Phyllodactylus martini* in allopatry versus sympatry

Prey	PC1|PrC	PC2|PrC	PC3|PrC
*Araneae*	−0.473|0.286	**0.818**|**0.387**	−5.72e−3|0.003
*Blattaria*	—	—	—
*Chilopoda*	−0.004|0.003	−0.011|0.005	7.04e−5|0.000
*Coleoptera*	**0.870**|**0.526**	0.411|0.194	2.05e−2|0.012
*Diptera*	0.036|0.022	−0.100|0.047	−3.28e−3|0.002
*Ephemeroptera*	−0.005|0.003	−0.014|0.007	1.93e−2|0.012
*Hemiptera*	−0.002|0.001	−0.019|0.009	−5.19e−2|0.031
*Hymenoptera*	−0.036|0.022	−0.265|0.125	0.651|0.390
*Isopoda*	−0.061|0.037	−0.037|0.018	2.35e−2|0.014
*Lepidoptera*	−0.023|0.014	−0.245|0.116	**−0.745**|**0.447**
*Orthoptera*	−0.100|0.061	−0.119|0.056	0.130|0.078
*Scorpiones*	−0.042|0.026	−0.072|0.034	−1.84e−2|0.011
% Variance explained	**16.59%**	**9.61%**	**5.98%**

Note

Top contributing components indicated in bold for each axis.

Abbreviations: PrC, Proportional contribution to PC axis.

**TABLE 3:** Principal component factor loadings by axis for invertebrate diet components of *Hemidactylus mabouia* in allopatry versus sympatry

Prey	PC1|PrC	PC2|PrC	PC3|PrC
*Araneae*	0.125|0.068	−5.14e−2|0.032	0.379|0.157
*Blattaria*	4.57e−2|0.025	−1.43e−2|0.009	1.10e−1|0.045
*Chilopoda*	−3.47e−18|0.000	−1.67e−16|0.000	−1.73e−17|0.000
*Coleoptera*	**−0.914**|**0.500**	4.50e−2|0.030	−0.153|0.063
*Diptera*	5.76e−2|0.032	−2.01e−2|0.012	0.162|0.067
*Ephemeroptera*	—	—	—
*Hemiptera*	3.61e−2|0.020	3.01e−2|0.019	4.49e−2|0.019
*Hymenoptera*	0.277|0.151	**0.734**|**0.454**	−0.471|0.194
*Isopoda*	0.222|0.122	−0.673|0.416	**−0.588**|**0.243**
*Lepidoptera*	0.120|0.066	−4.22e−2|0.026	0.473|0.195
*Orthoptera*	3.05e−2|0.017	−8.10e−3|0.005	4.26e−2|0.018
*Scorpiones*	1.11e−16|0.000	1.53e−16|0.000	−2.22e−16|0.000
% Variance explained	**19.60%**	**14.16%**	**12.04%**

Note

Top contributing components indicated in bold for each axis.

Abbreviations: PrC, Proportional contribution to PC axis.

### 3.2 Differences in morphology

**FIGURE 5:**
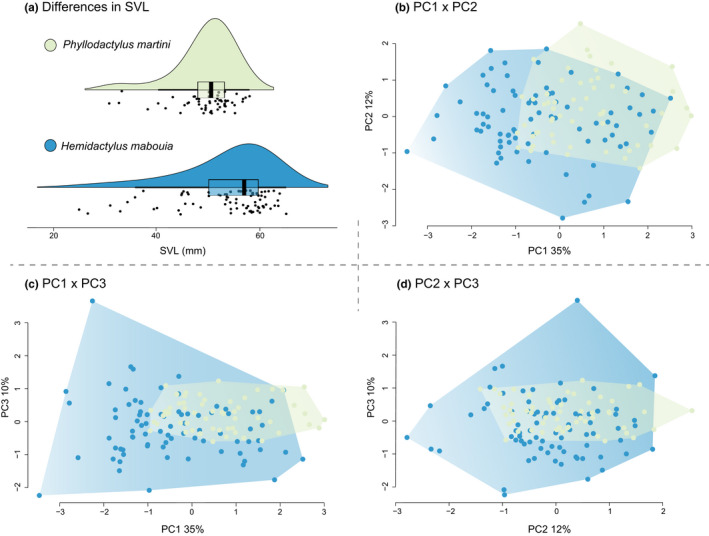
Analysis of morphometric traits. (a) Raincloud plots visualizing SVL differences between *Phyllodactylus martini* and *Hemidactylus mabouia*, depicting the frequency distribution through a rotated violin plot (top), box plot summary of quartiles (middle), and raw data (bottom) for each species. (b–d) Principal components analysis showing overlap of morphological traits between species. Principal component scores are visualized for each axis and species with background shading representing fitted convex hulls of the morphospace occupied by each species

We found a significant overall size difference between *H*. *mabouia* and *P*. *martini* (*F* = 10.61, *p* = .00143), with *H*. *mabouia* generally being larger (Figure 5a). Three axes of a principal components analysis (PCA) of morphological traits collectively capture 64.1% of the measured variation (PC1: 34.84%; PC2: 16.90%; PC3: 12.37%). PC1 largely captures differences in limb lengths (~39% total hind limb and 18% total front limb) and variation in the postorbital width (~24%). In contrast, PC2 mostly captures variation in cranial measurements with over 70% of the loadings belonging to a combination of head length (~29%), jaw length (~17%), temporalis width (~13%), and postorbital width (~13%). PC3 largely captured further variation in cranial morphology (Table 4). Visualization of these PC axes revealed a high degree of overlap between species, with *H*. *mabouia* occupying more morphospace overall. Between PC1 and PC2 (Figure 5b), the total morphospace occupancy based on the convex hull area [CHA] of *H*. *mabouia* was 64% larger (*H*. *mabouia* CHA = 18.370; *P*. *martini* = 11.140). Similarly, between PC1 and PC3 (*H*. *mabouia* CHA = 22.724; *P*. *martini* = 5.898; Figure 5c) and PC2 and PC3 (*H*. *mabouia* CHA = 16.532; *P*. *martini* = 5.474; Figure 5d), the CHAs of *H*. *mabouia* were larger. Results of our dataset resampling analyses support that these differences were not due to sample size differences alone (Figure S3) and that mean trait values (Table 5) vary between species. SVL was significantly correlated with all measured morphological traits and ANCOVA results further support significant differences between residual trait variations after accounting for SVL scaling between species for all traits (Table 6). The only exception to this general trend of a significant relationship between species identity and trait was head height (*F* = 3.232, *p* = .075, *q* = 0.075). These results were consistent whether non‐significant interactions were included in the analysis or not (Table S3). Tests for sexual dimorphism show no evidence for trait differences between male and female *P*. *martini* (Figures S4 and S5). In contrast, head width was significantly different between male and female *H*. *mabouia*, suggesting *H*. *mabouia* males have wider heads than females (Figure S4).

**TABLE 4:** Principal component factor loadings by axis for morphological traits of *Phyllodactylus martini* and *Hemidactylus mabouia*

Region	Trait	PC1|PrC	PC2|PrC	PC3|PrC
Cranial	Head height	−0.071|0.029	−0.094|0.038	**0.790**|**0.329**
	Head length	0.083|0.034	**−0.700**|**0.285**	−0.266|0.111
	Jaw length	−0.145|0.059	−0.407|0.166	−0.209|0.087
	Post orbital width	**0.581**|**0.235**	−0.307|0.125	0.195|0.081
	Temporalis width	0.177|0.072	−0.318|0.130	0.304|0.127
Limb	Thigh length	**−0.581**|**0.235**	−0.289|0.118	0.261|0.109
	Brachium length	−0.142|0.058	−0.218|0.089	−0.044|0.018
	Antebrachium length	−0.302|0.122	−0.108|0.044	−0.224|0.093
	Shin length	−0.386|0.157	0.012|0.005	0.107|0.045
% Variance explained		**34.84%**	**16.90%**	**12.37%**

Note

Trait values represent size‐corrected residuals from regressions to snout–vent length (SVL). Top contributing traits indicated in bold for each axis.

Abbreviations: PrC, Proportional contribution to PC axis.

**TABLE 5:** Raw measured morphological characters by species (in mm)

Region	Trait	*Hemidactylus mabouia* mean|N|SD	*Phyllodactylus martini* mean|N|SD
Cranial	Head height	5.95|79|1.33	5.66|57|0.74
	Head length	17.15|79|2.50	15.77|57|1.68
	Jaw length	9.94|79|1.37	8.92|57|0.98
	Postorbital width	5.56|79|1.23	5.85|57|0.94
	Temporalis width	10.77|79|1.84	10.12|57 |1.20
Limb	Thigh length	9.50|79|1.68	8.24|57|1.06
	Brachium length	4.79|79|0.99	4.02|57|0.64
	Antebrachium length	7.35|79|1.30	6.53|57|0.89
	Shin length	6.28|79|1.19	5.57|57|0.95
	Front:Hind Limb Ratio	0.23|79|0.07	0.23|57|0.08

Abbreviations: *N*, number of samples; SD, standard deviation.

**TABLE 6:** ANCOVA results testing the effect of snout–vent length (SVL), species, and their interaction on measured morphological characters

Region	Trait	*N*	df	log(SVL)/(*F*/*p*)/*q*	Species/(*F*/*p*)/*q*	Species:log (SVL)/(*F*/*p*)/*q*
Cranial	Head height	136	1	**358**.**09**/***/***	3.23/0.07/0.07	0.35/0.55/0.79
	Head length	136	1	**1298**.**73**/***/***	**101**.**84**/***/***	0.07/0.78/0.79
	Jaw length	136	1	**526**.**77**/***/***	**86**.**16**/***/***	0.18/0.66/0.79
	Postorbital width	136	1	**121**.**83**/***/***	**5.99**/*/*	0.07/0.78/0.79
	Temporalis width	136	1	**1151**.**42**/***/***	**32**.**45**/***/***	0.07/0.78/0.79
Limb	Thigh length	136	1	**307**.**27**/***/***	**59**.**16**/***/***	2.19/0.14/0.63
	Brachium length	136	1	**163**.**53**/***/***	**43**.**52**/***/***	**8.30**/**/*
	Antebrachium length	136	1	**464**.**95**/***/***	**54**.**76**/***/***	0.09/0.76/0.79
	Shin length	136	1	**140**.**64**/***/***	**20**.**94**/***/***	0.08/0.76/0.79

Note

Bolded values indicate significant effects. * stands for *p*‐values ranging from .05 to .01, ** for *p*‐values ranging from .01 to .001, and *** for *p*‐values <.001. The significance of *p*‐values, after adjusting for multiple comparisons, is shown as “*q*”.

Abbreviations: AN, number of samples; df, degrees of freedom; *F*, *f*‐statistic.

GLM analyses of the relationship between head size and hind limb length reveal largely concordant patterns regardless of which metric (head length or postorbital width) is used to quantify head size (Table 6, Figures S4 and S5). For both measurements, the top model (lowest AICc score) was the one containing a different intercept of the relationship between head size and limb length for the two species, but without a difference in slope (i.e., no interaction between species identity and the head size/SVL relationship). These top models also include no effect of sex on the relationship between head size and limb length, but in both cases the model that did include sex was also within or nearly within the set of credible models (deltaAIC of 1.52 for head length and deltaAIC of 2.2 for postorbital width).

## 4 DISCUSSION

The dominant hypothesis that explains the success of invasive *H*. *mabouia* populations is that they restrict access to food resources (Rocha et al., 2011; Williams et al., 2016), thereby promoting the extirpation of both native and even other non‐native gecko species (Meshaka, 2000; Short & Petren, 2012). Our study provides support for this hypothesis by demonstrating that *H*. *mabouia* consumes the same prey resources as *Phyllodactylus martini* in areas where these species co‐occur in Curaçao. Our analysis further identifies potential mechanisms for competitive advantage that may result in the disappearance of *P*. *martini* following the establishment of *H*. *mabouia*. We demonstrate larger sizes in feeding‐associated traits that may be advantageous for *H*. *mabouia* during the rapid forward propulsive locomotion associated with ambush predation as well as for capturing larger prey. Additionally, both stable isotopic and stomach contents analyses demonstrate that *H*. *mabouia* will readily consume vertebrate prey items that include *P*. *martini*, *Gonatodes antillensis* (the Venezuelan Coastal Clawed Gecko), and the non‐native blind snake *Ramphotyphlops braminus*. Given the ubiquity of *H*. *mabouia* throughout the neotropics, our results provide a new perspective for understanding the complexity of *Hemidactylus* spp. invasions.

### 4.1 Dietary overlap between *Hemidactylus mabouia* and *Phyllodactylus martini*

Our analyses demonstrate overlap of major invertebrate prey categories between *H*. *mabouia* and *P*. *martini* when the two species co‐occur (Figure 4; Figure S1). However, our analyses also reveal significant differences in diet between *P*. *martini* populations that are sympatric or allopatric with *H*. *mabouia*. In sympatry, the most common prey categories consumed by *P*. *martini* largely reflect common groups of invertebrates associated with human dwellings and artificial lighting in Curaçao (Dornburg et al., 2016). These categories are consistent with the diet of *H*. *mabouia* in other urbanizing areas (Bonfiglio et al., 2006; Drüke & Rödder, 2017; Iturriaga & Marrero, 2013), but contrast with the diet of *P*. *martini* in the dry mondi habitats of Curaçao. As such, dietary differences between populations of *P*. *martini* could reflect the movement of *P*. *martini* into a new foraging niche on human structures that places this species into direct contact and possible conflict with *H*. *mabouia*.

Urbanizing habitats have a pronounced effect on the foraging strategy of geckos, as the energetic cost of finding prey is reduced through utilization of artificial lights as a lure for attracting large prey resources (Gaston et al., 2013) that simultaneously confer a thermal advantage (Perry et al., 2008). This spatial clustering of food resources increases the probability of interaction and food resource competition between *H*. *mabouia* and *P*. *martini* and raises the question of whether *H*. *mabouia* has a morphological advantage for either capturing larger prey items or defending these aggregated food resource centers. We observed larger fragments of prey items such as roaches in the stomachs of *H*. *mabouia* in comparison with *P*. *martini*. Although partially digested fragments of invertebrate body parts prohibit further testing of whether *H*. *mabouia* is exploiting larger prey, our morphological analyses lend some insight that either of these hypotheses are possible. *H*. *mabouia* possesses overall larger body sizes, as well as larger heads, hind limbs, and other traits relative to *P*. *martini* (Figure 5; Tables 5 and 6). Increases in head height and head length are associated with increases in bite force and more efficient prey capture in geckos (Cameron et al., 2013; Massetti et al., 2017), as well as other lizard species (Dufour et al., 2018; Verwaijen et al., 2002). Functionally, this advantage is thought to arise by the combination of increasing space to accommodate increases in mandible adductor muscle sizes as well as changes in attachment angle that provide force advantages (Herrel et al., 2001). These changes in size and potential bite force poise *H*. *mabouia* to both acquire prey that may be more difficult for *P*. *martini* to capture, and better defend ambush sites from competitors. Indeed, the finding of *P*. *martini* as a prey item for *H*. *mabouia* suggests that such interactions could have very asymmetric fitness consequences. More behavioral observations in the field are warranted.

It is additionally possible that habitat‐specific changes in prey base are a major determinant of the spatial distribution and the interactions of *H*. *mabouia* and *P*. *martini* that could explain our observed differences in total prey count. For example, both high numbers of terrestrial isopods in some individual *H*. *mabouia* and a higher level of nematodes likely transmitted from arthropod vectors (Dornburg et al., 2019) in *P*. *martini* suggest local variation in prey. However, our results also reveal a broad degree of dietary niche overlap within each site and a consistent pattern of overlap between sympatric habitats. This broad dietary overlap in sympatry may reflect increased homogenization of the prey base, a phenomenon commonly associated with urbanizing areas (Bang & Faeth, 2011; Fenoglio et al., 2021). Investigating the changes in species composition of Curacao's ecological communities in response to urbanization represents an urgent research frontier. Such efforts will be crucial not only for gaining additional insights into the biology of these geckos but also for contextualizing shifts in the food web critical to forecasting the conservation needs of a wide range of taxa on this island.

### 4.2 The locomotor morphology of *Hemidactylus mabouia* and *Phyllodactylus martini*

It is well known that larger heads confer prey capture and bite force advantages to lizards (Cameron et al., 2013; Massetti et al., 2017). However, larger heads also come at a cost to locomotion as they negatively impact sprinting speed in lizards (Cameron et al., 2013; Lailvaux et al., 2019; Lopez & Martin, 2002). Our analyses suggest that increased hind limb lengths in both sexes of *H*. *mabouia* relative to *P*. *martini* may reflect the species partially mitigating a fundamental locomotor trade‐off (Tables 5 and 6; Figure S5). In lizards, longer hind limbs are often correlated with increased sprint speeds and forward propulsion (Bonine & Garland, 1999; Cameron et al., 2013; Winchell et al., 2018), thereby providing an advantage for an ambush predator such as *H*. *mabouia* relying on a combination of ambush and pursuit to capture prey. A similar increased scaling in hind limb lengths has been reported in *Hemidactylus frenatus* (Cameron et al., 2013) and our results suggest this is potentially a general feature of *Hemidactylus* locomotor morphology that allows these geckos to maintain sprinting speeds and prey capture advantages.

It is intuitively appealing to view the larger front limb size of *H*. *mabouia* relative to *P*. *martini* as an additional locomotor advantage for sprinting. However, recent work placing front limbs into the context of gecko locomotion models (Birn‐Jeffery & Higham, 2016; Zhuang & Higham, 2016) provides strong evidence that locomotor function is decoupled between fore and hind limbs. In contrast to hind limbs, which act as primary axes of propulsion, front limbs are primarily used for braking and downward locomotion (Birn‐Jeffery & Higham, 2016). As such, shorter front limbs shorten the swing time, the time needed to complete the swing phase of the gait cycle, thereby aiding in maintaining speed and stance in downward movements (Birn‐Jeffery & Higham, 2016). Quantifications of limb morphology across major lineages of geckos suggest shorter front limbs relative to hind limbs to be a hallmark of gecko locomotor morphology, with all species having between a 10 and 35% reduction in front limb proportions (Hagey et al., 2017), a finding consistent with our analysis of both *H*. *mabouia* and *P*. *martini* limb proportions (Table 5; Figure S5). Our finding of shorter front limbs relative to hind limbs in *H*. *mabouia* is consistent with expectations of selection for locomotion on steeply inclined surfaces such as walls that are coupled with large hind limbs for sprinting; but the significant negative scaling relationship between forelimb length and body size for *P*. *martini* also highlights the potential that there are additional major differences in locomotor mode and performance between these species.

Currently, the functional morphology and associated performance of *P*. *martini* remain little studied, as does that of *H*. *mabouia* in their native range in sub‐Saharan Africa. These studies are of particular importance as *H*. *mabouia* is increasingly found in non‐urban areas throughout its invaded range (Rocha et al., 2011), raising the question of which habitats offer the highest locomotor advantages to this competitor. While our study suggests that the vertical surfaces of dry forests in Curaçao provide habitat potentially suitable, if not advantageous, for *H*. *mabouia*, this species has yet to be found outside of urbanizing landscapes on that island. We speculate one potential reason for the absence of *H*. *mabouia* from the native bush habitat on Curaçao, and other similar desert habitats, is that all *Hemidactylus* geckos possess a basal toe pad system that may not be capable of successfully gaining traction on the loose, and dusty, rocky soil of the island (Russell & Delaugerre, 2017). Future analyses testing performance between and within *H*. *mabouia* and *P*. *martini* on different substrates may offer a particularly promising and exciting research frontier with high conservation applicability. For example, if *H*. *mabouia* is indeed a poor locomotor on sandy substrates, native gecko populations could be preserved integrating continual corridors of native semi‐arid and arid habitat into urban planning efforts. Such efforts would yield “enemy‐free” space and thereby increase the probability of the long‐term persistence of native gecko species such as *P*. *martini* (Cole et al., 2005).

## CONFLICT OF INTEREST

The authors declare no competing interests.

## AUTHOR CONTRIBUTION

**April Lamb:** Conceptualization (equal); Data curation (equal); Formal analysis (lead); Investigation (equal); Methodology (equal); Project administration (equal); Software (equal); Validation (lead); Visualization (equal); Writing – original draft (lead); Writing – review & editing (equal). **Catherine A. Lippi:** Data curation (equal); Investigation (equal); Methodology (equal); Writing – review & editing (equal). **Gregory J. Watkins‐Colwell:** Conceptualization (equal); Investigation (equal); Methodology (equal); Writing – review & editing (equal). **Andrew Jones:** Data curation (equal); Formal analysis (equal); Investigation (equal); Methodology (equal); Visualization (equal); Writing – review & editing (equal). **Dan L. Warren:** Conceptualization (equal); Formal analysis (equal); Investigation (equal); Methodology (equal); Software (equal); Validation (equal); Visualization (equal); Writing – review & editing (equal). **Teresa L. Iglesias:** Conceptualization (equal); Investigation (equal); Methodology (equal); Validation (equal); Writing – review & editing (equal). **Matthew C. Brandley:** Conceptualization (equal); Data curation (equal); Investigation (equal); Methodology (equal); Writing – review & editing (equal). **Alex Dornburg:** Conceptualization (equal); Formal analysis (equal); Funding acquisition (lead); Investigation (equal); Methodology (equal); Project administration (equal); Supervision (equal); Validation (equal); Visualization (equal); Writing – original draft (supporting); Writing – review & editing (equal).

## Supporting information

Appendix S1

## Data Availability

All data and code used to perform the analyses associated with this study have been archived on Zenodo (https://zenodo.org/badge/latestdoi/296632095).
