# Supplementary material for: Comparing the dietary niche overlap and ecomorphological differences between invasive Hemidactylus mabouia geckos and a native gecko competitor
Source: Ecol Evol. 2021 Dec 20;11(24):18719–32. doi: 10.1002/ece3.8401 (PMC8717282; doi:10.1002/ece3.8401)
Supplement: Supplementary file 1 — Appendix S1 [file ECE3-11-18719-s001.docx]

**Supplementary Materials**

*Ecology and Evolution*

**Comparing the dietary niche overlap and ecomorphological differences between *Hemidactylus mabouia* and *Phyllodactylus martini***

April D. Lamb*^1^, Catherine A. Lippi^2^, Gregory J. Watkins-Colwell^3^, Andrew Jones^4^, Dan Warren^5^, Teresa L. Iglesias^6^, Matthew C. Brandley^7,8^, and Alex Dornburg^1^

^1^ Department of Bioinformatics and Genomics, University of North Carolina, Charlotte, Charlotte, NC 28223

^2^ Quantitative Disease Ecology and Conservation (QDEC) Lab Group, Department of Geography, University of Florida, Gainesville, FL 32611

^3^ Division of Vertebrate Zoology, Yale Peabody Museum of Natural History, New Haven, CT 06520 USA

^4^ Department of Ecology and Evolutionary Biology, Yale University, New Haven, CT 06520 USA

^5^ Biodiversity and Biocomplexity Unit, Okinawa Institute of Science and Technology Graduate University, Okinawa Prefecture 904-0495, Japan

^6^ Animal Resource Section, Okinawa Institute of Science and Technology Graduate University, Okinawa Prefecture 904-0495, Japan

^7^ Section of Amphibians and Reptiles, Carnegie Museum of Natural History, Pittsburgh, PA 15213, USA

^8^ Powdermill Nature Reserve, Carnegie Museum of Natural History, Rector, PA, USA.

Contents

Table S1. Museum catalog numbers and collection locality [2](https://docs.google.com/document/d/14x0gLM5HSpRpFrXzxQVsnOSOM8-X1Snu/edit#heading=h.30j0zll)

Table S2.Relative frequency of prey stomach content items3

Table S3. ANCOVA results: SVL, species, and their interaction on measured morphological characters5

Fig. S1.Comparisons of dietary overlap between species 6

Fig. S2.Comparisons of dietary overlap between male and female *H. mabouia* 7

Fig. S3.Sampling procedure assessment 8

Fig. S4.Comparison of feeding-associated traits between species 9

Fig. S5.Locomotion-associated trait comparison between species 10

**Supplemental Table 1** | Museum catalog numbers and collection locality of *Phyllodactylus martini* and *Hemidactylus mabouia* specimens used. Abbreviations: CARMABI, Caribbean Research and Management of Biodiversity; YPM HERR, Division of Vertebrate Zoology Herpetology Collection (Reptiles), Peabody Museum of Natural History, Yale University.

| **Locality- Habitat Type**   ***Phyllodactylus martini Hemidactylus mabouia*** | | |
| --- | --- | --- |
| *Willemstadt-* Allopatric | --- | **YPM HERR** 18122, 18124, 18356, 18357, 18358, 18359, 18350, 18351, 18352, 18353, 18354**,** 18355, 18121, 18122, 18124 |
| *Saint Anna Bay* - Allopatric | --- | **YPM HERR** 18151, 18152, 18154 |
| *CARMABI*- Sympatric | **YPM HERR** 18437, 18435, 18436 | **YPM HERR** 18121, 18376, 18434 |
| *Lagun*- Sympatric | **YPM HERR** 18462, 18463, 18466, 18461, 18467, 18626 | **YPM HERR** 18464, 18470, 18478, 18475, 18474, 18472, 18471, 18465, 18468, 18473, 18476, 18469 |
| *Westpunt*- Sympatric | **YPM HERR** 17585, 17586, 17587, 17588, 17589, 17590, 17591, 17592, 17593, 17594, 17595, 17596, 17597, 17598, 17600, 17601, 17602, 17604, 17607, 18168, 18169, 18170, 18171, 18172, 18173, 18175, 18176, 18177, 18179, 18180, 18141, 18183, 18184, 18165, 18167, 18185, 18186, 18187, 18616, 18612, 18617, 18614, 18645, 18646, 18622, 18623, 18611, 18619 | **YPM HERR** 18133, 18134, 18135, 18136, 18137, 18140, 18127, 18142, 18143, 18144, 18632, 18630, 18633, 18631, 18638, 17557, 17563, 17564, 17565, 17566, 17567, 17568, 17569, 17570, 17571, 17572, 17573, 17575, 17576, 17577, 17578, 18127, 18127, 18129, 18130, 18131, 18132, 18133, 18134, 18135, 18136, 18137, 18138, 18139, 18140, 18142, 18143, 18144, 18145, 18146, 18149, 18150, 18151, 18152, 18154, 18155, 18631 |
| *Shete Boca*- Allopatric | **YPM HERR** 18342, 18343, 18344, 18345, 18345, 18347, 18348, 18349, 18629, 18625, 18624, 18627, 18628, 18626 | --- |

**Supplemental Table 2 |** Relative frequency of prey stomach content items across all sampled individual *Hemidactylus mabouia* (total items = 72) and *Phyllodactylus martini* (total items = 115)*.* P = pooled samples, S= sympatric sites, A = allopatric sites, n = number of prey items, N = number of individual lizards, m = mean number of prey items per individual (n/N).

| ***Hemidactylus mabouia Phyllodactylus martini*** | | |
| --- | --- | --- |
| **Prey Relative Frequency Relative Frequency**  **P,n,N,m \| S,n,N,m \| A,n,N,m P,n,N,m \| S,n,N,m \| A,n,N,m** | | |
| Arachnida | 0.14, 10, 8, 1.13 \| 0.11, 6, 5, 1.20 \| 0.24, 4, 3, 1.33 | 0.19, 22, 19, 1.16 \| 0.19, 17, 14, 1.21 \| 0.19, 5, 5, 1 |
| Scorpiones | --- | 0.05, 6, 5, 1.20 \| 0.06, 5, 4, 1.25 \| 0.04, 1, 1, 1 |
| Araneae | 0.14, 10, 8, 1.25 \| 0.11, 6, 5, 1.20 \| 0.24, 4, 3, 1.33 | 0.14, 16, 14, 1.14 \| 0.14, 12, 10, 1.20 \| 0.15, 4, 4, 1 |
| Chilopoda | --- | 0.01, 1, 1, 1 \| 0.01, 1, 1, 1 \| --- |
| Insecta | 0.49, 35, 34, 1 \| 0.44, 24, 24, 1 \| 0.65, 11, 10, 1.10 | 0.58, 67, 57, 1.18 \| 0.66, 58, 48, 1.21 \| 0.33, 9, 9, 1 |
| Blattaria | 0.03, 2, 2, 1 \| --- \| 0.12, 2, 2, 1 | --- |
| Coleoptera | 0.13, 9, 9, 1 \| 0.15, 8, 8, 1 \| 0.06, 1, 1, 1 | 0.24, 27, 23, 1.17 \| 0.27, 24, 20, 1.20 \| 0.11, 3, 3, 1 |
| Diptera | 0.06, 4, 4, 1 \| 0.09, 4, 4, 1 \| --- | 0.08, 9, 7, 1.29 \| 0.10, 9, 7, 1.29 \| --- |
| Ephemeroptera | --- | 0.01, 1, 1, 1 \| 0.01, 1, 1, 1 \| --- |
| Hemiptera | 0.04, 3, 3, 1 \| 0.02, 1, 1, 1 \| 0.176, 3, 2, 1.5 | 0.02, 2, 2, 1 \| 0.02, 2, 2, 1 \| --- |
| Hymenoptera | 0.10, 7, 7, 1 \| 0.07, 4, 4 \| 0.18, 3, 3 | 0.06, 7, 7, 1 \| 0.08, 7, 7, 1 \| --- |
| Lepidoptera | 0.10, 7, 7, 1 \| 0.09, 5, 5, 1 \| 0.12, 2, 2, 1 | 0.10, 11, 7, 1.57 \| 0.11, 10, 6, 1.67 \| 0.04, 1, 1, 1 |
| Orthoptera | 0.03, 2, 2 \| 0.04, 2, 2, 1 \| --- | 0.09, 10, 10, 1 \| 0.06, 5, 5, 1 \| 0.19, 5, 5, 1 |
| Isopoda | 0.28, 20, 6, 3.33 \| 0.33, 18, 4, 4.50 \| 0.12, 2, 2, 1 | 0.09, 10, 6, 1.67 \| 0.02, 2, 2, 1 \| 0.30, 8, 4, 2 |
| Skin Shed | 0.01, 1, 1, 1 \| 0.19, 1, 1, 1 \| --- | 0.01, 1, 1, 1 \| 0.01, 1, 1, 1 \| --- |
| Vertebrata | 0.04, 3, 3, 1 \| 0.06, 3, 3, 1 \| --- | --- |
| *Gonatodes antillensis* | 0.01, 1, 1, 1 \| 0.19, 1, 1, 1 \| --- | --- |
| *Phyllodactylus martini* | 0.01, 1, 1, 1 \| 0.19, 1, 1, 1 \| --- | --- |
| *Ramphotyphlops braminus* | 0.01, 1, 1, 1 \| 0.19, 1, 1, 1 \| --- | --- |
| Other | 0.01, 1, 1, 1 \| 0.19, 1, 1, 1 \| --- | 0.11, 13, 13, 1 \| 0.09, 8, 8, 1 \| 0.19, 5, 5, 1 |

**Supplemental Table 3 |** ANCOVA results testing the effect of snout-vent length (SVL), species, and their interaction on measured morphological characters with non-significant interactions removed. Bolded values indicate significant effects. * stands for *P*-values ranging from 0.05 to 0.01, ** for *P*-values ranging from 0.01 to 0.001 and *** for *P*-values smaller than 0.001. The significance of *P*-values, after adjusting for multiple comparisons, are shown as “q”.

| **Trait** | **N** | **df** | **log(SVL) / (F/P) / q** | **Species / (F/P) / q** | **Species:log(SL) / (F/P) / q** |
| --- | --- | --- | --- | --- | --- |
| Post Orbit Width | 136 | 1 | **122.68 / *** / ***** | **6.02 / * / *** | --- |
| Temporalis Width | 136 | 1 | **1159.48 / *** / ***** | **32.68 / *** / ***** | --- |
| Head Length | 136 | 1 | **1307.8 / *** / ***** | **102.6 / *** / ***** | --- |
| Jaw Length | 136 | 1 | **530.02 / *** / ***** | **86.69 / *** / ***** | --- |
| Head Height | 136 | 1 | **359.84 / *** / ***** | 3.248 / 0.0738 / *** | --- |
| Brachium Length | 136 | 1 | **163.54 / *** / ***** | **43.521/ *** /** 0.07 | **8.302 / ** / **** |
| Antebrachium Length | 136 | 1 | **468.16 / *** / ***** | **55.14 / *** / ***** | --- |
| Thigh Length | 136 | 1 | **304.54 / *** / ***** | **58.63 / *** / ***** | --- |
| Shin Length | 136 | 1 | **141.61 / *** / ***** | **21.09 / *** / ***** | --- |


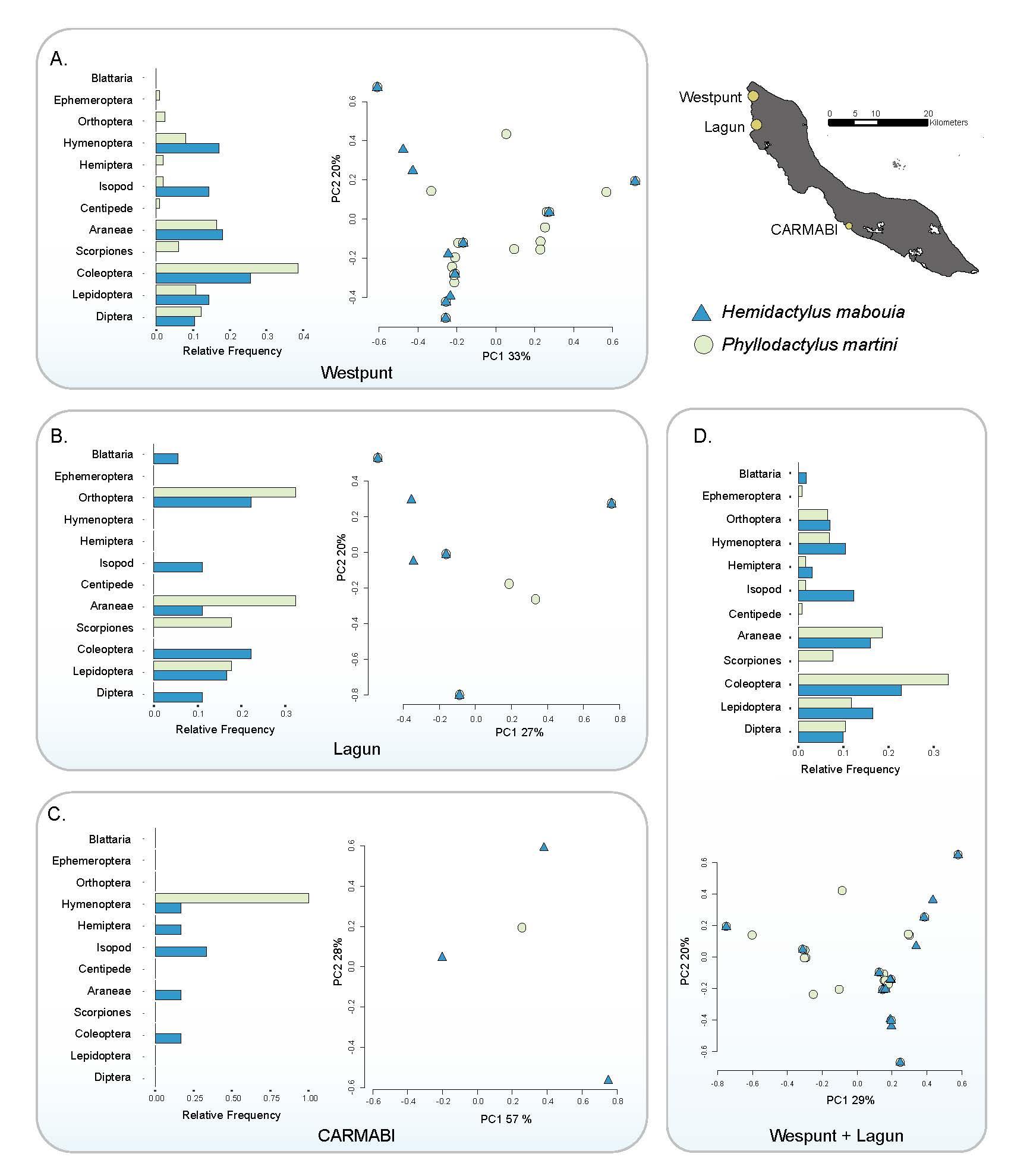


**Supplemental Figure 1** | Comparisons of dietary overlap in areas where *Hemidactylus mabouia* (blue) and *Phyllodactylus martini* (green) co-occur: (A) Westpunt, (B) Lagun, (C) CARMABI, (D) Westpunt and Lagun pooled. Left side of each panel depicts the relative frequencies of prey item percentages encountered within individuals. The right side of each panel depicts a visualization of the PC analysis with shadings that correspond to convex hulls. In all cases there is large overlap between species. Top-right inset: map of sampling locations


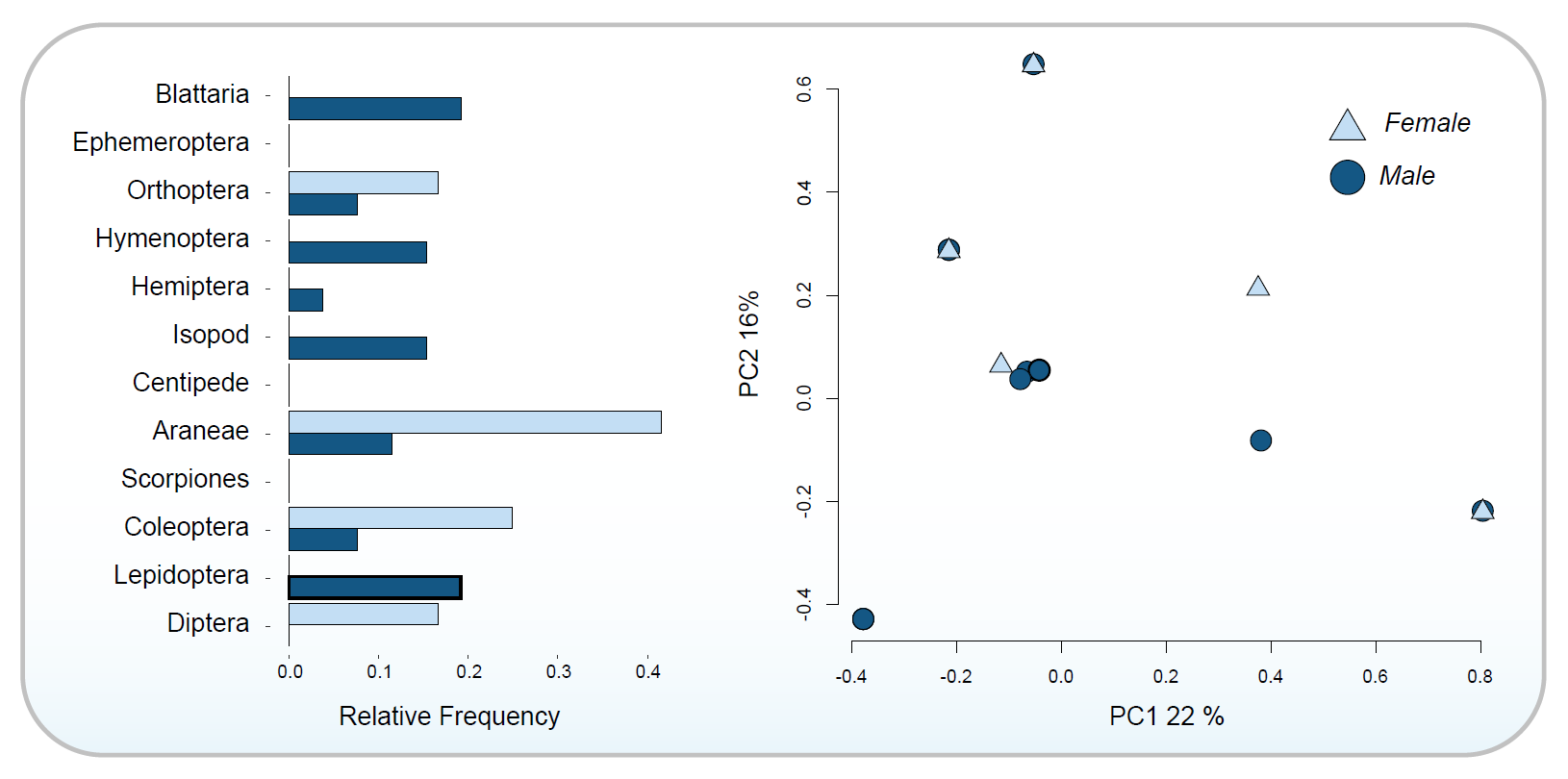


**Supplemental Figure 2 |** Comparisons of dietary overlap between male (dark blue) and female (light blue) *Hemidactylus mabouia.* Left panel depicts the relative frequencies of prey item percentages encountered within individuals. Right panel depicts a visualization of the PC analysis with shadings that correspond to convex hulls.


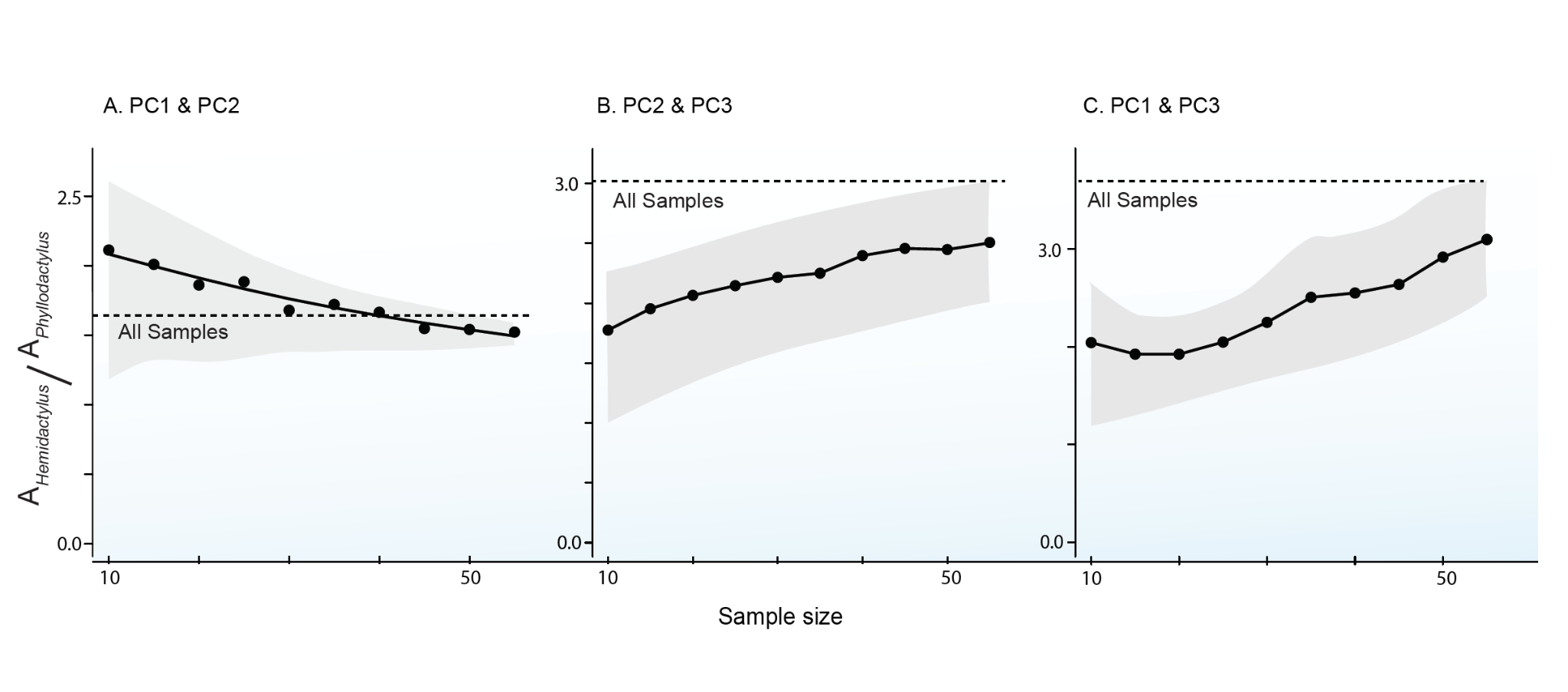


**Supplemental Figure 3 |** Results from a resampling procedure assessing differences in morphospace occupation between *Phyllodactylus martini* and *Hemidactylus mabouia* under different levels of individual sampling. Y axis represents the ratio of morphospace area (A) between *H. mabouia* and *P. martini*. X axis indicates the number of individuals drawn from our dataset at random for (A) PC1 & PC2; (B) PC2 & PC3; and (C) PC1 & PC3. Gray area represents the 25 and 75% quantiles of the resampled morphospace differences, interpolated between sample size replicates. Dots represent the mean of the area ratio for each sample size replicate, with a spline interpolation (solid line) between points. Dotted line indicates the area ratio quantified when all samples are included in the morphospace (Main Text Figure 4).

**
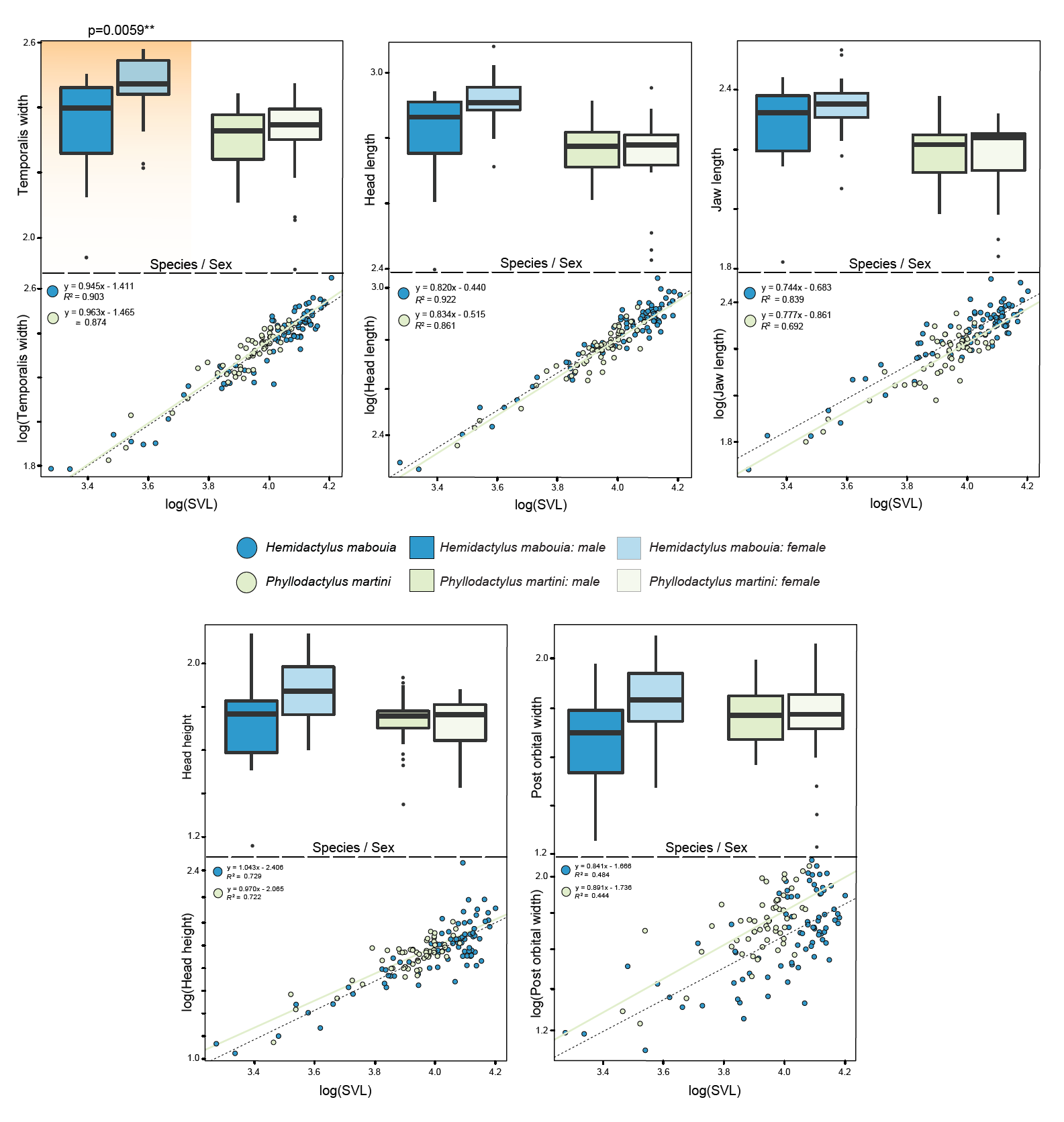
**

**Supplemental Figure 4 |** Feeding-associated trait comparison between *Phyllodactylus martini* (green) and *Hemidactylus mabouia* (blue). Differences in morphological trait size between sexes and species (top) and results from simple linear regression (SLR) between snout to vent length (SVL) and morphological traits (bottom). Traits (from right to left, top to bottom): temporalis width, head length, jaw length, head height, post-orbital width. All lengths in log(mm). Box plot shadings correspond to species + male/female designations in the figure. Orange shading in the temporalis width plot indicates a statistically significant difference in male and female temporalis width.

**
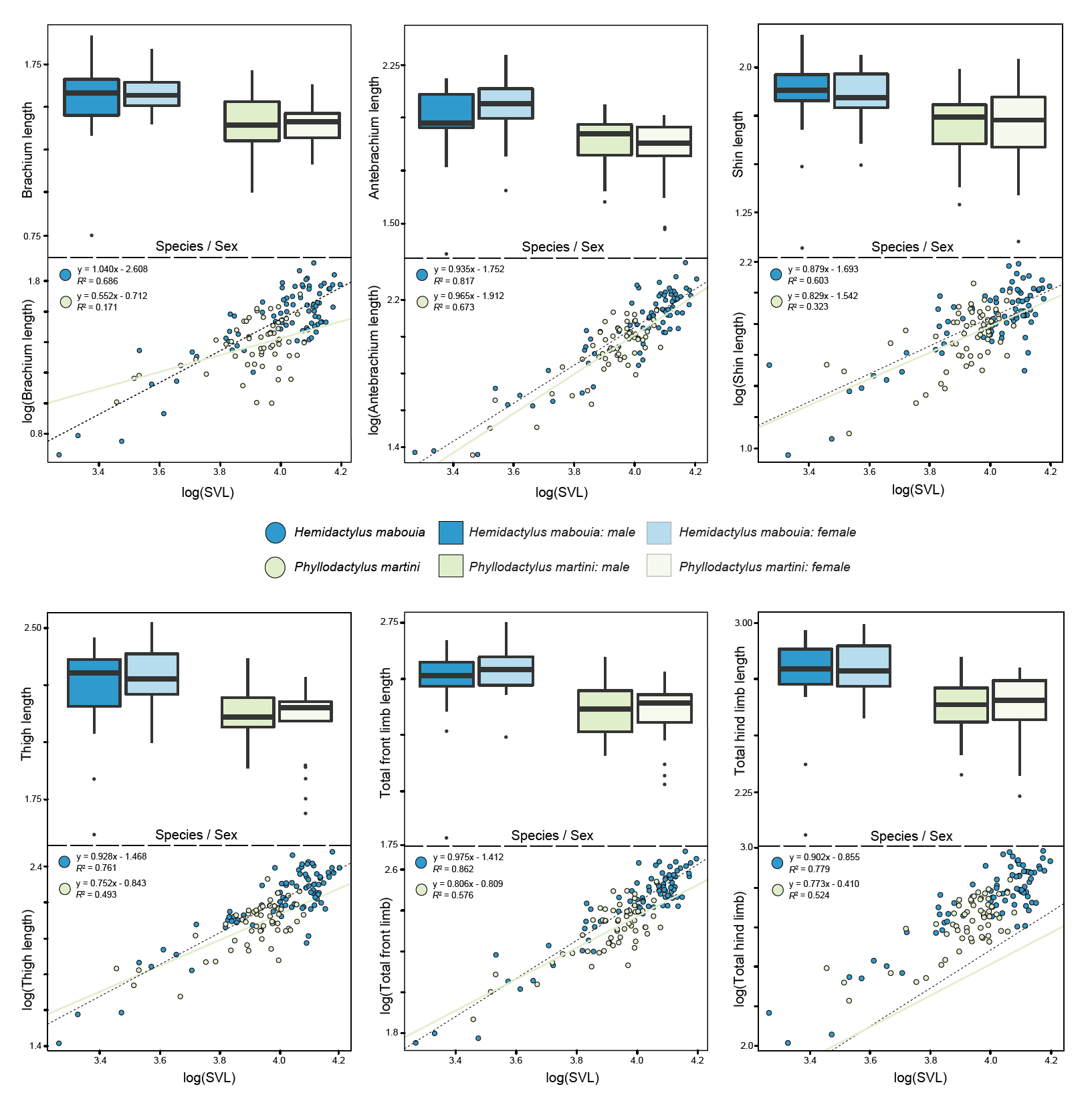
**

**Supplemental Figure 5** | Locomotion-associated trait comparison between *Phyllodactylus martini* (green) and *Hemidactylus mabouia* (blue). Differences in locomotion-associated morphological trait size between sexes and species (top) and results from simple linear regression (SLR) between snout to vent length (SVL) and morphological traits (bottom). All lengths in log(mm). Traits include (from left to right, top to bottom) brachium length, antebrachium length, thigh length, shin length, total upper limb length (brachium+antebrachium), and total lower limb length (thigh+shin). Box plot shadings correspond to species + male/female designations in the figure.
